# Comparison of marginal microleakage of metal copings cemented with three luting cements

**DOI:** 10.4317/jced.59343

**Published:** 2022-03-01

**Authors:** Keyla Vargas-Belón, Katya Chambilla-Torres, Marco Sánchez-Tito

**Affiliations:** 1 Private practice, Tacna, Peru; 2 Facultad de Ciencias de la Salud, Universidad Privada de Tacna, Tacna, Peru

## Abstract

**Background:**

To evaluate the marginal microleakage in single metal copings cemented with zinc phosphate, glass ionomer, and resin-modified ionomer.

**Material and Methods:**

An experimental in vitro study was carried out. The calculation of the sample was determined using the G*Power software; 15 premolars were considered per group. The teeth were carved considering a preparation with a chamfer-type shoulder with an angulation of 6°. Working models were obtained, where the metallic copings were made. Cementation was carried out with zinc phosphate (Prothoplast), glass ionomer (Ketac-Cem Easymix), and resin-modified ionomer (Relyx™ Luting 2) cements. The samples were immersed in 2% methylene blue solution for 24 hours. The microleakage measurement was carried out with a 40x stereo microscope at four measurement points (vestibular, lingual, mesial, and distal). In order to compare the microleakage values, the ANOVA test was carried out, followed by the Scheffé test. A significance level of 5% was adopted.

**Results:**

The zinc phosphate cement showed the highest values of marginal microfiltration (109.28 ± 51.27 µm) followed by the resin-modified ionomer cement (102.63 ± 45.07 µm) and the ionomer of glass (98.64 ± 39.18 µm), although these differences were not statistically significant (*p*>0.05).

**Conclusions:**

Zinc phosphate, glass ionomer, and resin-modified ionomer cements exhibited similar properties to prevent marginal microfiltration in unitary metal copings.

** Key words:**Cementation, dental leakage, dental restoration failure.

## Introduction

Porcelain-metal crowns have been used to restore decayed teeth for the past few decades. Ceramic crowns have gained popularity due to their superior esthetics and continue to be a treatment option in many clinical situations (1).

Several complications affect the survival of crowns in the mouth. These complications can be classified in general terms as biological, related to the patient, and techniques used for cementation (2). Some authors include endodontic complications, chipping and fracture of the porcelain, tooth fracture, periodontitis, and secondary caries at the tooth-material interface (3,4). The latter phenomenon is one of the main reasons for the failure of crown restorations (5). The key factors to ensure crowns’ longevity and clinical success are fracture resistance and marginal adaptation (6).

Cementation is essential for the long-term prognosis of crowns, as it seals the dead space (cement space), provides retention, prevents microleakage and secondary caries (7).

Various types of cements are currently available, from traditional cements such as zinc phosphate cement to resin-based or resin-modified cements. Zinc phosphate cement has been used in dentistry since the 1880s; it has a successful track record supported by clinical evidence, but due to its high solubility and low adhesion, it has high *in vitro* microleakage scores compared to other cements; despite this, this cement ensures the survival of crowns in the mouth, especially metal-ceramic and metal crowns (8-10). In the case of glass ionomer cement, its ability to adhere to the tooth structure and release fluoride is recognized, which is why its use has increased in recent decades. In addition, it has low solubility and disintegration values compared to other cements, thus reducing the percentage of microleakage (11). Over time, its composition underwent several modifications; the addition of resin to its composition allowed the uptake and release of calcium, fluoride, and phosphate ions in reaction to changes in pH in the oral environment; this bioactivity allowed better chemistry and bonds between the cement and dentin, thus reducing microleakage, improving durability and remineralization of the tooth (7).

Although there is no ideal luting agent that meets all the expected requirements, it is essential to consider the factor of microleakage when choosing one of these materials. Previous studies have analyzed the degree of marginal sealing of crowns concerning the type of cement used. Behnaz *et al*. showed that resin-modified cements have lower microleakage than glass ionomer and zinc phosphate cements, the latter having the highest microleakage values (12). Al-Haj *et al*. observed that glass ionomer cement had the highest microleakage compared to resin-modified cement and resin-based cement (13). Satyendra *et al*. studied the behavior of resin and resin-modified glass ionomer cements and showed that the resin-based cement produced less microleakage (14).

The objective of this study was to compare marginal microleakage in single-unit metal copings cemented with zinc phosphate, glass ionomer, and modified ionomer.

## Material and Methods

-Design and sample calculation

An *in vitro* experimental study was performed. The calculation of the sample was determined using the G*Power program. An effect size of 0.5, a significance level of 0.05, and a power of 0.8 were considered. Fifteen samples were estimated for each group. Prior to the execution of the study, authorization was obtained from the Ethics Committee of the Faculty of Health Sciences of the Universidad Privada de Tacna under registry no. 014-2020-FACSA/UPT.

-Sample preparation

Forty-five healthy premolars, extracted for orthodontic reasons, collected from various private dental centers in the city of Tacna, Peru, were used. The teeth were immersed in 2.5% sodium hypochlorite for 24 h to eliminate soft tissue debris and washed copiously with running water (13). Test tubes were made by immersing the teeth in Vitacryl self-curing acrylic (New Stetic, Antioquia, Colombia) up to 1 mm from the amelocemental junction. Then, the teeth were prepared, considering a chamfer-type shoulder preparation with an angulation of 6° (15), verified with a parallelogram (Bioart, Sao Paulo, Brazil). Coarse and fine-grained round-tipped truncated cone-shaped diamond burs (MDT, Israel) were used for the preparation. Subsequently, impressions of each test tube were taken with Zeta plus Oranwash - L condensation silicone (Zhermack, Badia Polesine, Italy) using polyvinyl chloride (PVC) tubes as specimens. The impressions were vacuum cast to obtain working models with type IV Resinrock plaster (WhipMix, Kentucky, USA). A Fornax -T induction furnace (BEGO, Bremen, Germany) was used to make the metal copings.

-Cementation and marginal seal measurement

**Figure 1:**
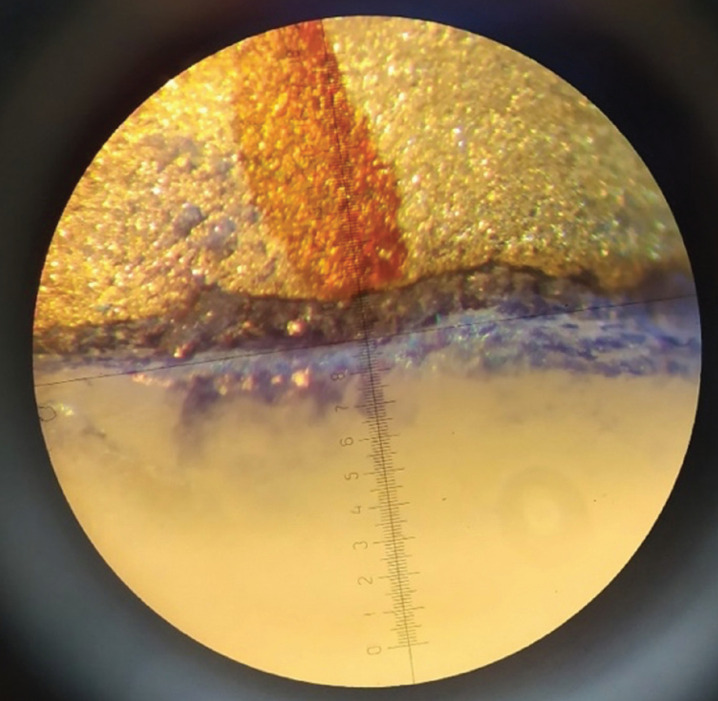
Measurement of microleakage at one of the sample reference points.

Prior to cementation, the sealing of the copings was verified using an explorer and fluid silicone technique. In the test tubes where misalignment of the copings was found, the manufacturing process was repeated. Samples were randomly assigned into three groups depending on the type of cement used for cementation: Prothoplast Zinc Phosphate (Laboratorios SL S.A., Buenos Aires, Argentina), Ketac-Cem Easymix glass ionomer (3M-ESPE, Neuss, Germany) and Relyx™ Luting 2 resin-modified ionomer (3M-ESPE, Saint Paul Minnesota, USA). The cements were prepared according to their manufacturers’ recommendations. Once set, the samples were immersed in 2% methylene blue solution for 24 hours (17). Microleakage was measured with a 40x stereomicroscope (Carl Zeiss Light Microscopy, Göttingen, Germany) using four measuring points: vestibular, palatal, mesial, and distal (Fig. 1).

-Data analysis

Data analysis was performed with the statistical program (SPSS- Statistical Package for the Social Sciences) version 25.0. The assumptions of normality and homogeneity of variances were checked with the Shapiro Wilk and Levene tests, respectively. The data was analyzed for the averages of each measurement point, and additionally, to this, the overall average for each cement was analyzed. The ANOVA test followed by the Scheffé test for multiple comparisons of marginal microleakage measurements was used to evaluate the difference between measurements. The significance level was adjusted to 5%.

## Results

Table 1 shows the average marginal microleakage produced by the cements. Glass ionomer cement showed the lowest microleakage values (98.64±39.18 µm), although no significant differences were observed with the other cements (*p*>0.05).

**Table 1:**
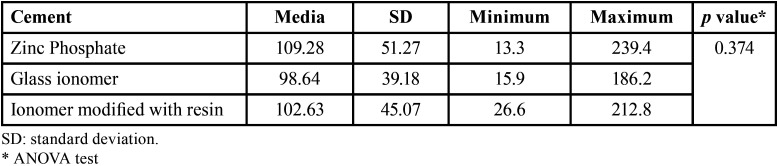
Average microleakage in cemented copings with the three cements (µm).

Table 2 highlights that in the case of zinc phosphate cement, a higher microleakage was observed on the lingual side with values of 124.13±54.76 µm; in the case of glass ionomer cement, the highest values were observed on the distal side with 114.38±36.17 µm, while in the case of resin-modified ionomer cement the mesial side was the one that obtained the highest microleakage values with 118.81±36.75 µm. However, no significant statistical differences were observed between the groups when the ANOVA test followed by the Scheffé test was performed (*p*>0.05).

**Table 2:**
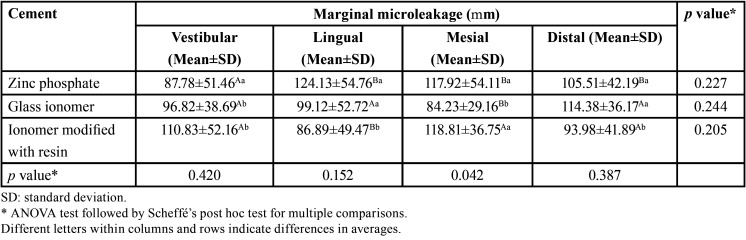
Microleakage in the cemented copings with the three cements for each measuring point (µm).

**Figure 2:**
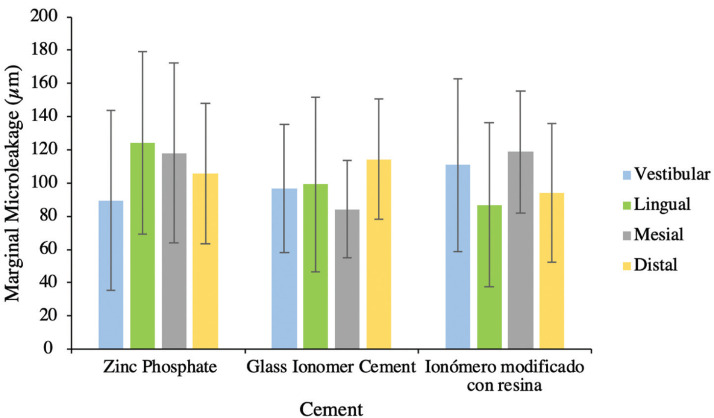
Marginal leakage error bars for cemented copings with the three cements per measurement point.

The Figure 2 shows a higher marginal microleakage on the vestibular side for the resin-modified ionomer cement (110.83±52.16 µm), while for the lingual side it was the zinc phosphate cement (87.78±51.46 µm), for the mesial side, it was again the resin-modified ionomer cement (118.81±36.75 µm) and for the distal side the glass ionomer cement (114.38±36.17 µm).

## Discussion

The appropriate selection of a luting agent is a relevant decision for the success of fixed restorations. The presence of marginal gaps and microleakage can affect their long-term permanence in the mouth (14,16). In the literature, the marginal sealing capacity of different cements used in the crown cementation process has been studied in order to demonstrate which one avoids or decreases marginal microleakage.

Ebadian *et al*. (12) studied the microleakage of restorations cemented with RelyX™ Ultimate, RelyX™ Unicem, GC Gold Label, and Hoffmann cements, finding that Hoffmann zinc phosphate cement obtained higher microleakage values (5.00±2.000 mm) followed by GC Gold Label glass ionomer cement (2.71±1,976 mm) and finally by the resinous cements RelyX™ Unicem (2.14±1.952 mm) and RelyX™ Ultimate (0.86±1.215 mm), considering the nature of the cements, these results are similar to those found in the present study, where the zinc phosphate cement presented higher microleakage versus the glass ionomer cement.

Parameswari *et al*. (17) observed that Harvard’s zinc phosphate® cement had the highest microleakage values (82.6±3.64 µm) followed by Multilink® resinous cement (57.6±3,435 µm) while Fuji’s GC glass ionomer cement® had the lowest values (28.6±5,413 µm). The study by Eftekhar *et al*. (18) showed that Fleck’s zinc phosphate cement obtained the highest microleakage results (3.32±0.70 mm), followed by G-Cem™ resin cement (2.08±1.10 mm), Fuji Plus® resin-modified glass cement (0.92±0.53 mm) and finally Panavia™ F2.0 resin cement (0.64±0.78 mm). Al-Haj *et al*. (13) observed that Ketac Cem Aplicap™ glass ionomer cement obtained higher microleakage results (0.67±0.27 mm) while lower values were recorded for Rely X™ Unicem (0.30±0.30 mm). Satyendra *et al*. (14) showed that Relyx Lut U 200 resin cement obtained lower microleakage values (64.04 ±7.44 µm) when compared to Relyx Lut 2 resin-modified ionomer cement (74.26 ± 7.67 µm). These results suggest that zinc phosphate cement has higher marginal microleakage than glass ionomer and resin-modified ionomer cements; this characteristic was similar in the present study, although the differences between the level of microleakage between the types of cements were not statistically significant.

The difference in microleakage between cements reported in previous studies could be explained by the various components that can affect their properties. Zinc phosphate cement is a type of acid-base cement used mainly as a cementing agent, known for its clinical use in the routine cementation of metal-supported crowns and bridges (17). It is characterized by a low pH of 2 to 4 when the powder is mixed with the liquid, the pH increases during setting to reach neutrality in one to two days (8,9). The initial acidic environment will cause an increase in solubility, causing slow erosions due to a combination of abrasions and dissolutions, resulting in microleakage points. Despite this, it has one of the highest survival rates with respect to crowns in the mouth (10).

Type I glass ionomer is exclusive for cementation. It is characterized by superior adhesion to dental tissues and a low coefficient of exothermic expansion, similar to dentin, acting as a thermal insulator, thus reducing possible future microleakage (19). In addition, although this cement has an initial acid pH, it neutralizes quickly, so it would not influence later problems (11). On the other hand, the resin-modified ionomer is a variation of conventional glass ionomer, to which methacrylate has been added. It is characterized by lower sensitivity to humidity and better mechanical behavior, thus reducing marginal microleakage and improving its adhesion to dentin. In addition, it has a much-improved resistance to dissolution compared to previous cements (7,20).

The findings of previous studies (12-14,17,18) suggest that, depending on the nature and type of cement, the results are similar concerning the property of sealing crowns at the marginal level. The standardization of the test specimens is an essential factor to ensure the correct interpretation of the data; other factors such as the degree of angulation, storage conditions, type of tooth, application of thermocycling techniques could also influence the results, considering the inherent limitations of *in vitro* studies to reproduce the conditions of the oral environment. It is necessary to consider future studies that can include and control as many of these variables as possible (21).

Some of the limitations of the present study were the accessibility of only one type of microscope for microleakage analysis; more sophisticated techniques such as confocal microscopy may be recommended for linear measurements using photosensitive pigmenting agents. In addition, the use of artificial aging methods for microleakage measurement could generate more accurate values by allowing a closer approximation to oral conditions (21,22).

## Conclusions

In conclusion, in this research, and under the methodology employed, it was observed that there are no statistically significant differences in marginal microleakage in metal copings cemented with zinc phosphate, glass ionomer, and resin-modified ionomer cements.
